# Practical Hints on the Treatment of Females in the Advanced Stage of Pregnancy

**Published:** 1818-07-01

**Authors:** Richard Moulson


### *Practical Hints on the Treatment of Females in the advanced Stage of Pregnancy.*

By Richard Moulson, M. D.

On Tuesday, the 15th of April, 1817, I was requested, by a physician accoucheur, to examine the body of a young woman who had died of hæmorrhage the preceding Sunday night, a very few hours after delivery. She had been attended, during her confinement, by a midwife; the labour terminated naturally, if we except the hæmorrhage which succeeded. This young married woman, being a domestic in one of the first families in the west end of the town, every attention was paid to her by her mistress, who, as soon as she learnt that her servant was in danger, immediately sent for Dr. ——. Dr. —— arrived just in time to witness the fatal event, and judging that this was a case of uterine hæmorrhage, was anxious to know the real state of things, and, with some difficulty, obtained permission to examine the body.

On the day above-mentioned, I went to the house, in the neighbourhood of Portman-square, in company with Dr. ——. Having laid the corpse upon the table, I remarked, that I scarcely ever saw such fine proportion exemplified in an individual. The subject appeared about 22 years of age, rather *embonpoint*, and possessing the most placid countenance; nor was there the least discolouration of any part of the body.

The abdomen was very tense, and did not pit upon pressure. It appeared fully as prominent as it must have done previous to the birth of the child. This appearance certainly seemed to strengthen the opinion that she had fallen a victim to uterine hæmorrhage.

Upon laying aside the parietes of the abdomen, the omentum consisted merely of membrane, very little fat being attached to it, and it could scarcely be called diseased; the stomach was healthy: the liver was much paler than natural, very large, and contained, within the right lobe, a tubercle of a beautiful white colour, of the consistence of soap. The intestines were very much distended with air, and here and there had small black patches upon them. At the sigmoid flexure of the colon, mortification had commenced, which extended along the rectum, including in its ravages the whole of the cellular membrane covering the psoæ muscles: the left iliac region was perfectly black, and upon separating the peritoneal covering of the psoæ and iliac muscles, coagula of blood were found, upon the removal of which, the finger readily passed through into the vagina. Here the mortification was so extensive, that it was impossible to discover from what vessel or vessels the hæmorrhage had proceeded: all in the neighbourhood of this disease seemed to have suffered the common lot of annihilation. Certain as it was, that the disease alone was sufficient to have caused death, still the profuse flooding succeeding immediately to the delivery, hastened that period which allowed of an investigation into its real cause.

The quantity of fæces within the colon was so great as to feel like one solid substance, (indeed I could only compare it to a large Bologna sausage), and evidently was the cause of the mortification. The uterus was perfectly healthy and properly contracted: the right Fallopian tube was also healthy, as was the right ovarium; but the left Fallopian tube was perfectly black, having partaken of the mortification from the parts with which it was connected, yet the disease did not extend to the uterus nor left ovarium. A slight effusion of serum, of a dark colour, was likewise found within the cavity of the abdomen. Time, and the eye of an inquisitive relative, would not allow of a more minute investigation. This woman had for many days, previous to her delivery, complained of great pain on the left side of the belly.

A few days after the examination of the above case, I was desired to see a woman who had been safely delivered by a midwife, on the Surrey side of Westminster bridge, two or three days before I was called in. My attendance was required to allay most distressing *after-pains.* I learnt that this woman had taken two or three doses of opening medicines, but without the desired effect. It was noon when I saw her, and she declared, that if she had had one stool that morning she had had forty. Luckily I saw what she had designated by the name of a stool, and which I was convinced was only the forerunner of what remained behind. Her abdomen was very tense, and extremely pained with only the pressure of the bed clothes; her milk was gone; she complained of great pain in the right iliac region, to which I ordered twelve leeches to be immediately applied. Gruels would not remain upon her stomach, and her pulse was not less than 125. At that time I had several cases of puerperal fever under my care, yet this woman's symptoms, in some respects analogous, were in others very different, and made me conclude, from the previous dissection, and from taking every circumstance into consideration, that her present alarming situation depended upon fæces lodged in the intestines. I immediately ordered fl. ʒj. tinc. opii to be given to her in an enema, to allay the irritation in the intestines; in the evening, finding that it had produced the desired effect, I gave her a bolus composed of hydrargyri submuriatis gr. v. jalapæ pulv. gr. xv. with two table spoons full of a purgative mixture composed of infus. sennæ and sulphat. magnesiæ, every three hours. The next morning, when I visited her, she was quite a different woman, entirely free from pain, had an enlivened countenance, could bear pressure with the hand upon the abdomen, which was now flaccid; and, to use her own words, she "had filled three chamber-pots with fæces since last I visited her." By attention to the bowels, and light nourishing gruels, this woman was up in a week.

The few remarks I have to make are, upon the necessity of paying strict attention to the bowels during pregnancy. That this necessity may be evident to most practitioners I do not mean to doubt; but that it is acted upon in the manner it ought to be, I positively deny. Will that accoucheur dare to say, that he has acted conscientiously to his patient, whom he is in the habit of attending, during her accouchements, when he finds his hand that supports the perinæum to be clogged with fæces at the time when the head is passing through the os externum? That many of the most difficult natural labours are owing to the state of the rectum impeding the free passage of the child's head, every rational practitioner will agree. Then, why is the injurious custom of giving opium by the mouth, to allay spurious pains, still persevered in, when a good enema will do much better, and at the same time dislodge any offending matters in the lower part of the intestines, before the head has descended so low that an enema can be of little or no use. I have seen a woman literally in torments upon the labour bed with what are called spurious pains, when upon examination, the os uteri was not dilated more than a sixpence, yet the pains were continual, racking, and exhausting the powers of the woman. An enema under such circumstances has brought away a chamber-pot full of fæces, sleep has been induced, and in three or four hours the labour has terminated happily both to mother and child.

Medicine, when judiciously administered, may be given at any period of pregnancy; and I do not hesitate to say, that had the unfortunate subject of the above-related dissection had her bowels attended to previous to her confinement, she might, deo volente, have been alive now; for a better formed pelvis never was seen. If the French are not so fortunate in curing diseases by their lavements, &c. they undoubtedly prevent many from occurring.

I am well aware, that the confidence of many practitioners is sadly abused, in the treatment of female complaints, by that false delicacy which is instilled into the minds of the most amiable part of the creation, by what is called genteel education. Most complaints referable to the alimentary canal and urinary organs are now-a-days confined principally to the bosoms of the unfortunate sufferers, and to make mention of them would be considered indelicacy in the extreme. If, "*orandum est ut sit mens sana in corpore sano;*" why are not the first principles of health made familiar and impressive to the minds of the rising generation, that under the most painful and natural processes of life, disease and danger may be warded off, and parturition made a blessing instead of its being in many instances a curse. It is needless for me to ask whence originated nervous diseases, as they are called, with their accompanying panaceas, bilious and anti-bilious pills, disgracing our daily prints? To enlarge upon the subject just hinted at, would here be out of place, but I seriously believe that if the man who couching his words in plain and comprehensive language, suitable to the delicacy of the sex to which he addresses himself, was to point out the serious evils that may be avoided by a strict attention to certain rules and regulations on the subject hinted above, he would be conferring an everlasting kindness, and be acknowledged the friend and benefactor to the female sex.

*Halifax, Yorkshire, 25th March,* 1818.

